# Antibodies Against *Pseudomonas aeruginosa* Alkaline Protease Directly Enhance Disruption of Neutrophil Extracellular Traps Mediated by This Enzyme

**DOI:** 10.3389/fimmu.2021.654649

**Published:** 2021-03-31

**Authors:** Chendi Jing, Chenghua Liu, Yu Liu, Ruli Feng, Run Cao, Zhangchun Guan, Bo Xuan, Yaping Gao, Qi Wang, Nana Yang, Yuanfang Ma, Lefu Lan, Jiannan Feng, Beifen Shen, Hui Wang, Yanyan Yu, Guang Yang

**Affiliations:** ^1^ State Key Laboratory of Toxicology and Medical Countermeasures, Beijing Institute of Pharmacology and Toxicology, Beijing, China; ^2^ Department of Infectious Diseases, Peking University First Hospital, Beijing, China; ^3^ Department of Clinical Laboratory, Peking University First Hospital, Beijing, China; ^4^ Joint National Laboratory for Antibody Drug Engineering, School of Basic Medical Sciences, Henan University, Kaifeng, China; ^5^ Department of Clinical Laboratory, Peking University People’s Hospital, Beijing, China; ^6^ Department of Molecular Pharmacology, Shanghai Institute of Materia Medica, Chinese Academy of Sciences, Shanghai, China

**Keywords:** *Pseudomonas aeruginosa*, alkaline protease, alkaline protease inhibitor, neutrophil extracellular traps, antibody-dependent enhancement

## Abstract

Extracellular traps released by neutrophils (NETs) are essential for the clearance of *Pseudomonas aeruginosa*. Alkaline protease (AprA) secreted by *P. aeruginosa* negatively correlates with clinical improvement. Moreover, anti-AprA in patients with cystic fibrosis (CF) can help identify patients with aggressive forms of chronic infection. However, the mechanism underlying the clinical outcomes remains unclear. We demonstrated that *aprA* deficiency in *P. aeruginosa* decreased the bacterial burden and reduced lung infection. AprA degraded NET components *in vitro* and *in vivo* but did not affect NET formation. Importantly, antibodies induced by AprA acted as an agonist and directly enhanced the degrading activities of AprA. Moreover, antisera from patients with *P. aeruginosa* infection exhibited antibody-dependent enhancement (ADE) similar to that of the antibodies we prepared. Our further investigations showed that the interaction between AprA and the specific antibodies might make the enzyme active sites better exposed, and subsequently enhance the recognition of substrates and accelerate the degradation. Our findings revealed that AprA secreted by *P. aeruginosa* may aggravate infection by destroying formed NETs, an effect that was further enhanced by its antibodies.

## Introduction

*Pseudomonas aeruginosa* is an opportunistic gram-negative bacterium that is ubiquitous in the environment. Normally, serious *P. aeruginosa* infections are prevalent in immunocompromised individuals, including patients with neutropenia, severe burns, cystic fibrosis (CF), and chronic obstructive pulmonary disease (COPD) (1, 2). As a consequence, *P. aeruginosa* is a leading cause of nosocomial infections and is one of the most common pathogens associated with ventilator-associated pneumonia, which contributes to a high morbidity and mortality in critically ill patients (3). In recent years, therapeutic options have become increasingly limited due to the emergence of multidrug-resistant *P. aeruginosa* strains, highlighting the fact that this bacterium has become a serious clinical problem (4).

*P. aeruginosa* secretes a number of extracellular proteases that allow it to escape the host immune attack, such as elastase and alkaline protease (AprA) (5, 6). AprA is a 50-kDa zinc metalloprotease secreted by the type I secretion system. In addition, *P. aeruginosa* encodes alkaline protease inhibitor (AprI), a specific inhibitor of AprA, that is predicted to be a periplasmic protein and protects self-proteins from degradation by AprA (7). Previous studies have demonstrated that AprA is detected in the majority of human patients with CF infected with *P. aeruginosa* and that AprA levels correlate inversely with clinical improvement (8, 9). However, the role of AprA in the pathogenesis of *P. aeruginosa* infection and its precise mechanism remain obscure.

Neutrophils are the most abundant innate immune cells in humans and play a critical role in controlling bacterial infections. In addition to phagocytosis and degranulation, neutrophils can also form neutrophil extracellular traps (NETs) to combat pathogens (10, 11). NETs are extracellular fibrous structures composed of decondensed chromatin, histones, and granular-derived antimicrobial peptides such as cathepsin G, myeloperoxidase (MPO), and neutrophil elastase (NE) (11, 12). It has been demonstrated that *P. aeruginosa* is able to induce a robust release of NETs from human neutrophils (13, 14). In addition, extensive neutrophilic infiltration and numerous NETs can be detected in most diseases associated with *P. aeruginosa* lung infection (15–17).

In the present study, we found that AprA degraded the components of NETs and thereby ensured effective immune evasion by *P. aeruginosa*. Moreover, we discovered that antibodies against AprA acted as an agonist and enhanced the degradation activity of AprA, contributing to the pathogenesis of *P. aeruginosa* infection.

## Materials and Methods

### Experimental Design

Trial experiments done previously were used to determine sample size with adequate statistical power. Participants are randomly allocated to each independent variable group. The investigators were blinded to group allocation during data collection and analysis.

### Bacterial Strains and Growth Conditions

The *Escherichia coli* strains BL21 and DH5α were purchased from Novagen. PAO1 were kindly provided by professor Lefu Lan (Department of Molecular Pharmacology, Shanghai Institute of Materia Medica, Chinese Academy of Sciences). *E.coli* cells and *P. aeruginosa* cells were grown in Luria-Bertani (LB) medium at 37°C for 12 h with shaking at 220 rpm.

### Construction of PAO^aprA-^ Strain

To construct the aprA mutant strain (PAO^aprA-^), a SacB-based strategy (18) was employed. Briefly, the upstream fragment of the intended deletion was amplified from PAO1 genomic DNA using the specific primers (D-aprA-up: F 5’- CCCAAGCTTCGGAGAACACCTACAACCAG -3’, R 5’- CGGGATCCGCAGTTATCGGCCAGATCAG -3’), while the downstream fragment was amplified with specific primers (D-aprA-down: F 5’- CGGGATCCCCAGGACAAGATCGACCTGT -3’, R 5’- GGAATTCATGATCGGTCGCTTCGTGGT -3’). The two PCR products were digested and then cloned into vector pEX18Ap, yielding pEX18Ap::*aprA*UD. Subsequently, a *ca*. 1.8 kb gentamicin resistance cassette cut from pPS858 with *Bam*HI was cloned into pEX18Ap::*aprA*UD, yielding pEX18Ap::*aprA*UGD. The resultant plasmids were electroporated into Wild Type PAO1 with selection for gentamicin resistance. Colonies were screened for resistance to gentamicin and sucrose and sensitivity to carbenicillin, which typically indicates a double-cross-over event and thus of gene replacement occurring. The deletion of *aprA* in PAO1 was further confirmed by PCR and gene sequencing.

### Construction of PAO^aprA-^ (pAK1900-*aprA*) Strain

To construct the *aprA* rescued strain-PAO^aprA-^ (pAK1900-*aprA*), PCRs were performed to amplify *aprA* from PAO1 genomic using the specific primers (*aprA*: F 5’-AACAAGCTTGTGTCAGTTTGGACTTCGGC-3’, R 5’-TTTGGATCCAACCGCGCCCGTGTCAGCGCG-3’). The PCR products were cloned into the vector pAK1900, then the resultant plasmids were electroporated into PAO^aprA-^ with selection for gentamicin resistance. Colonies with dual resistance to gentamicin and carbenicillin were selected on LB agar plates. The PAO^aprA-^ (pAK1900-*aprA*) strain were further confirmed by PCR and gene sequencing.

### Expression and Purification of Recombinant Proteins

The *aprA*, *aprI* and *flagellin* were amplified by PCR, using the genome of PAO1 with the specific primers (*aprA*: F 5’-CGCGGCAGCATATGGACGAATTGGTCAATGGC-3’, R 5’-CACCACCACTCGAGTCAGACGACGATGTCGG-3’; *aprI*: F 5’-CACCACCAGCTAGCAGTCTGATTCTTCTCAGCG-3’, R 5’-CACCACCACTCGAGCTAGGGCGTCGCGCGGCGCAGC-3’; *flagellin*: F 5’-CATGACCAGCTAGCATGGCCCTTACAGTCAACACGAAC-3’, R 5’-TGGTCATGCTCGAGTTAGCGCAGCAGGCTCAGGACCG-3’). The PCR products were cloned into the expression vector pET-28 (a), which were subsequently expressed in *E.coli* (BL21) and purified using Ni-NTA agarose (GE Healthcare, 17-5318-01) according to manufacturer’s instructions. The site-directed mutagenesis of AprA (ΔAprA) plasmid was constructed by the pET-28 (a)-AprA plasmid with specific primers (F1 5’-GTATGTCAGCGATATCTACGCGCTGGGCGCGTTCAGCGCCTTTTCCGC-3’, R1 5’-AAGGCGAACGCCGCACCGCCGACCGCCGCGCTGAAGTTGCCGAAGGTCA-3’; F2 5’-ATCAACGCGAGCTACAGCGCCAACGTCAATC-3’, R2 5’-TGTAGCTCGCGTTGATCAGGTACCAGGATTGC-3’; F3 5’-GGTGCGGCGTTCGCCTT-3’, R3 5’-GTAGATATCGCTGACATACTTCCAC-3’) *via* a Fast MultiSite Mutagenesis System (Transgen, Cat. No#FM201-01). The mutant was screened and further confirmed by DNA sequencing. The plasmid with desired mutants was then transformed into *E.coli* (BL21) for protein expression as described above. Protein concentrations were quantified by use of the bicinchoninic acid protein assay, and proteins were stored at -80°C until use.

### Extraction and Purification of Histones

HL-60 cells (5x10^6^/ml) were collected and lysed in 1ml hypotonic lysis buffer for 30 min at 4°C. After centrifugation, the samples were resuspended in 400 μl 0.4N H_2_SO_4_ and incubated at 4°C for at least 30 min. Then the histones in the supernatant were precipitated with TCA and washed twice with ice-cold acetone. Protein concentrations were quantified as described above.

### Production of Ra-Anti-AprA (Rabbit Antibodies Against AprA) and Hu-Anti-AprA (Human Antibodies Against AprA)

Ra-anti-AprA was induced after the administration of AprA in Female New Zealand White rabbits after three immunizations with 1mg of AprA mixed with complete Freund’s adjuvant (first immunization) and incomplete Freund’s adjuvant (second and third immunization). Then, the antibody titer and specificity of the sera were analyzed by ELISA and Western blotting. Ra-anti-AprA was purified using an immune-affinity column which was prepared by conjugating protein A according to the manufacturer’s instructions.

Hu-anti-AprA was isolated from a phage display antibody library using the standard procedure (19). In brief, AprA was coated on Nunc-immunotube (Thermo Scientific Nunc^®^, 444202) at 4°C overnight. Then phages (10^12^ plaque-forming units [PFU]/ml) were incubated with AprA for 1h, unbound phages were removed. The bound phages were eluted using 0.1M Gly-HCl (pH 2.2). *E. coli* TG1 cells were infected with the eluted phages. The amplified phages were then subjected to the next round of panning. After three rounds of panning, single colonies were randomly selected and further screened by ELISA. The sequences of the specific phage clones binding to AprA were analyzed.

### Human Neutrophils Isolation and NETs Formation

Human neutrophils were isolated from peripheral blood of healthy volunteers using standard density gradient as described previously (12). Cells were resuspended in complete medium (RPMI 1640 with 2% heat-inactivated human serum), 2x10^5^ neutrophils per well were seeded on the bottom of a cell culture dish and stimulated with PAO1, PAO^aprA−^, or PAO^aprA-^ (pAK1900-*aprA*) (MOI = 5, 10, and 50) for 3 h or with PAO1, PAO^aprA−^, or PAO^aprA-^ (pAK1900-*aprA*) (MOI = 50) for 30 min, 90 min, and 180 min.

### Measurement of ROS Production

ROS production was measured by dichlorofluorescin diacetate (DCFH-DA, Beyotime). Briefly, neutrophils were incubated with 10 μM DCFH-DA at 37°C for 30 min, cells were then washed and stimulated by different strains of *P. aeruginosa* (PAO1, PAO^aprA-^, PAO^aprA-^ (pAK1900-*aprA*), MOI=50), PMA (100 nM) or were left unstimulated. Finally, samples were analyzed by flow cytometry (BD).

### Measurement of NETs-Mediated Bacterial Killing

Human neutrophils (2x10^5^ per well) were resuspended in RPMI containing 2% heat-inactivated human serum and seeded into 96 well plates. After stimulated with PMA (100 nM, Sigma) for 30 min at 37°C, the samples were treated with the phagocytosis inhibitor cytochalasin D (10 μg/ml) and different concentrations of AprA (0, 10 μg/ml) or AprI (0, 100 μg/ml). After incubation for 2 h at 37°C, PAO1 were added to the neutrophils at MOI of 2. Then the plates were centrifuged for 10 min at 800 g and followed by incubation at 37°C for 90 min. Finally, the sample suspensions were serially diluted and plated on LB plates to determine the surviving bacterial. The result was normalized to bacterial growth without neutrophils under the same conditions.

### Murine Pneumonia Model

Specific pathogen free BALB/c mice (8-week-old females) were inoculated intranasally with different strains of *P. aeruginosa* (PAO1, PAO^aprA-^, PAO^aprA-^ (pAK1900-*aprA*), 5x10^6^ colony forming units (c.f.u.) per mouse) under anesthesia. The body weight was recorded at baseline, 6 h and 24 h post-challenge. Mice were sacrificed at different time points (at 6 h, 24 h and 48 h) post-challenge for further analyzes. Lungs were harvested and homogenized in phosphate-buffered saline plus 0.1% Triton X-100 under sterile conditions, serial dilutions of lysate were plated on LB plates to determine the bacterial load at 6h post-infection.

### *In Vivo* Treatment With Neutrophil Elastase Inhibitor (NEi)

The experiment was performed as previously described with some modifications (20). Briefly, mice were injected intraperitoneally with neutrophil elastase inhibitor (50 μg per mouse, GW311616A, AxonMedChem, Groningen, Netherlands) or PBS 12 h before *P. aeruginosa* infection and every 12 h post-challenge until final analyzes. Mice were sacrificed for NETs detection and lung bacterial burden evaluation at 6 h or 24 h post-challenge.

### Flow Cytometry

To analyze the neutrophil recruitment in the airways, BALF was collected by lavaging the lung with 1 ml PBS in tubes containing EDTA. After centrifugation, acquired cells were resuspended in flow cytometry buffer, followed by incubation with fluorochrome-conjugated anti-mouse antibodies against CD11b (Biolegend), Gr-1 (Biolegend) for 30 min at RT. Data were collected *via* flow cytometry (BD) and analyzed using FlowJo software (TreeStar).

### Immunofluorescence Confocal Microscopy

To analyze NETs formation *in vitro*, cells were fixed in 4% paraformaldehyde and permeabilized in 0.1% Triton X-100 prior to blocking with 5% Normal Donkey serum for 1 h at room temperature (RT). The samples were incubated with primary antibodies (goat anti-human/mouse myeloperoxidase/MPO [10 μg/ml Catalog # AF3667], rabbit anti-histone H3 (citrulline R2+ R8 + R17) [1:200 ab5103]) overnight at 4°C. For MPO and Cit-H3 staining, tetramethylrhodamine (TRITC)-conjugated goat anti-rabbit (1:100) and FITC-conjugated Donkey anti-goat antibody (1:100) were added for 1 h at RT, nuclei were stained with DAPI (1:5000) for 10 min at RT. The labeled cells were analyzed with a Carl Zeiss LSM 510 laser scanning confocal microscope.

Immunofluorescence staining of AprA expression and NETs formation *in vivo* was performed as previously described (21). Briefly, lungs were collected 6 h and 24 h after PA infection and fixed 48 h with 10% formalin. Lung tissues were embedded in paraffin and 5-μm-thick sections were cut for staining. After deparaffinization and rehydration, these sections were boiled for antigen retrieval, then permeabilized in 0.5% Triton X-100 prior to blocking with 4% Normal Donkey serum for 1 h at room temperature (RT). For detection of AprA, samples were stained with Ra-anti-AprA antibody, followed by a secondary tetramethylrhodamine (TRITC)-conjugated goat anti-rabbit antibody. For detection of NETs, lung sections were stained with antibodies described above. The samples were imaged and analyzed by a laser scanning confocal microscope. The immunofluorescence images were quantified using NIH ImageJ analysis software (ImageJ), according to previously established procedures (22).

### Western Blotting

After collection of human neutrophils and murine lung tissues (6 h and 24 h post-challenge), the samples were homogenized in RIPA lysis buffer containing protease inhibitor cocktails on ice. An equal amount of protein per sample was subjected to 12% SDS-PAGE and the proteins were blotted onto a Hybond-enhanced chemilumescent nitrocellulose membrane, which were then incubated with the primary antibodies (anti-human/mouse myeloperoxidase/MPO (AF3667, R&D), anti-histone H3 (citrulline R2 + R8 + R17) (ab5103, abcam), anti-histone H2A (ab177308, abcam), anti-histone H2B (ab52599, abcam), anti-histone H3 (ab1791, abcam), anti-histone H4 (ab10158, abcam), anti-His (HT501, Transgen Biotech), Ra-anti-AprA, Hu-anti-AprA), then subsequently with appropriate HRP-conjugated secondary antibodies (goat anti-rabbit IgG, goat anti-mouse IgG; rabbit anti-goat IgG,; goat anti-human IgG, Jackson ImmunoResearch Laboratories). Equal loading was confirmed by probing for β-actin (HC201, Transgen Biotech).

### Lung Histology

Lung tissues were routinely processed for paraffin embedding and were sectioned at 5-μm-thick for hematoxylin-eosin staining at 48 h post-infection. Histology was scored for intraluminal infiltrate, peribronchial infiltrate and alveolar involvement using a scoring system that described previously (23). At least ten representative, non-overlapping fields from a single lung section were independently and blindly evaluated by two pathologist.

### ELISA

AprA protein (10 μg/ml) was coated onto 96-well plates overnight. After washing and blocking, 100 μl of serially diluted Ra-anti-AprA, Hu-anti-AprA, and P1 through P5 were added to the wells respectively. Bound antibodies were detected with HRP-conjugated goat anti-rabbit IgG or HRP-conjugated goat anti-human IgG (Jackson ImmunoResearch Laboratories).

### Antibody Dependent Enhancement Assay

To study the antibodies role *in vitro*, antibodies (Ra-anti-AprA, Hu-anti-AprA, P1 through P5) were serial diluted and mixed with AprA or ΔAprA for 30 min at RT. Then the mixture were added to the samples (NETs, flagellin) and further incubated for 2 h at 37°C. After incubation, the samples were analyzed using a laser scanning confocal microscope or western blotting as described above.

To evaluate the antibodies role *in vivo*, mice were injected intraperitoneally with Hu-anti-AprA (100 μg per mouse) or control-IgG 24 h before PAO1 infection. At 6 h post-challenge, mice were sacrificed and the bacterial load in lungs was calculated as described above.

### Participants

Five patients with *P. aeruginosa* infection were recruited from Peking University People’s Hospital. Clinical features were obtained from the medical record database of Peking University People’s Hospital. The characteristics of these patients were presented in **Table S1**, healthy blood donors were served as controls.

### Statistical Analysis

All experiments were repeated at least three times and the data were presented as the mean ± SD unless noted otherwise. Data were evaluated using a two-tailed Student’s t test or Mann-Whitney test. A P-value of less than 0.05 was considered to be statistically significant. GraphPad Prism software was used for statistical analyzes.

### Ethics Approval and Consent to Participate

This study was approved by the ethics committee of the Peking University People’s Hospital (2019PHB077-01) and have been performed in accordance with the ethical standards as laid down in the 1964 Declaration of Helsinki and its later amendments or comparable ethical standards. Informed consent was not needed due to that the medical records and patient information were anonymously reviewed and collected in this study. All animal experimental protocols of the study are in accordance with the national guidelines for the use of animals in scientific research “Regulations for the Administration of Affairs Concerning Experimental Animals” and were approved by the Animal Care and Use Committee of Beijing Institute of Pharmacology and Toxicology.

## Results

### AprA Is Involved in the Lung Infection Caused by *P. aeruginosa*

**Figure 1:**
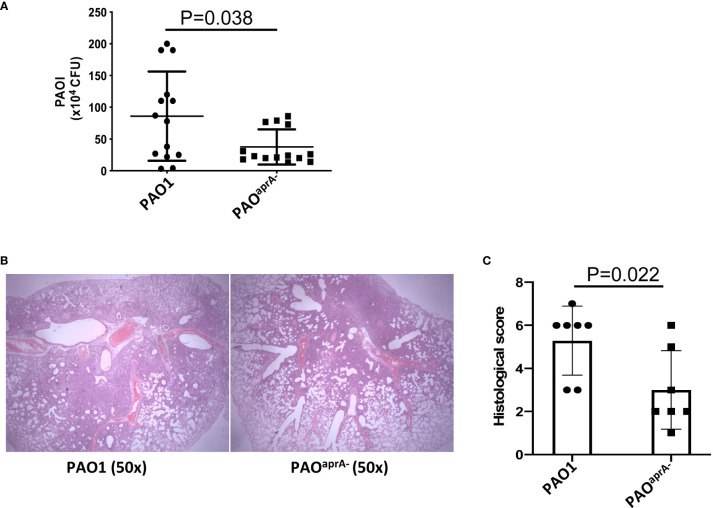
AprA is involved in the lung infection caused by *P. aeruginosa*. **(A)** Bacterial burden in the lung tissues of the indicated groups of mice. BALB/c mice (n = 14 per group) were inoculated intranasally with PAO1 or PAO^aprA-^ (5×10^6^ c.f.u. per mouse), respectively. The number of bacteria in lung tissues was evaluated at 6 h post-challenge. **(B)** Histological examination of lung tissues. Lung specimens were collected 48 h post-challenge and stained with hematoxylin-eosin. **(C)** Histopathology scores of lung tissues (n=7 per group). All data are representative of three independent experiments. Data in **(A, C)** are represented as mean ± SD. Significant differences between groups in **(A, C)** were evaluated using Mann-Whitney test.

To identify the role of AprA in the pathogenesis of *P. aeruginosa*, we developed a murine pneumonia model of bacterial infection through the intranasal inoculation of 5 × 10^6^ c.f.u. of different strains of *P. aeruginosa*, including a wild-type parental PAO1 and an *aprA* deletion mutant PAO^aprA−^. As expected, the number of bacteria in the lungs of mice in the PAO^aprA−^ group was lower than that in the parent strain (PAO1) group at 6 h post-challenge (**Figure 1A**). Histopathological examination of the lungs also showed that the PAO^aprA−^ group exhibited attenuated tissue damage compared to that of the PAO1 group at 48 h post-challenge (**Figure 1B**), this phenomenon was also confirmed using a quantitative scoring system (23) (**Figure 1C**). Moreover, the weight loss post-infection for the mice in the PAO^aprA−^ group tended to be less than that of the PAO1 group, although it did not reach a statistically significant difference (**Figure S1**).

### Deficiency of *aprA* Increased NET Levels *In Vivo*

To further explore the mechanism of AprA enhancement of *P. aeruginosa* pathogenesis, we generated an *aprA*-rescue strain of PAO^aprA−^ using the plasmid pAK1900-aprA. Compared with that in PAO1, the expression level of AprA was absent in PAO^aprA−^, with expression level being recovered in PAO^aprA-^ (pAK1900-*aprA*) (**Figure S2A**, **B**). Moreover, we also demonstrated that compared with PAO1 and PAO^aprA-^ (pAK1900-*aprA*), the enzymatic activity of AprA was significantly reduced in the supernatant of PAO^aprA-^ (**Figure S2C**).

Given that *P. aeruginosa* is able to induce robust infiltration of neutrophils during lung infections and neutrophils are considered to play an essential role in the clearance of *P. aeruginosa* (24, 25), we determined whether neutrophil levels were altered for the different *P. aeruginosa* strains in the murine pneumonia model. Female BALB/c mice (n = 10 per group) were challenged with a sub-lethal dose of PAO1, PAO^aprA−^, or PAO^aprA-^ (pAK1900-*aprA*) (5 × 10^6^ c.f.u. per mouse). Bronchoalveolar lavage fluid (BALF) was collected from the mice 6 h post-challenge and neutrophil numbers were evaluated by flow cytometry. There was no significant difference among the three groups of mice (**Figure S2D**).

**Figure 2:**
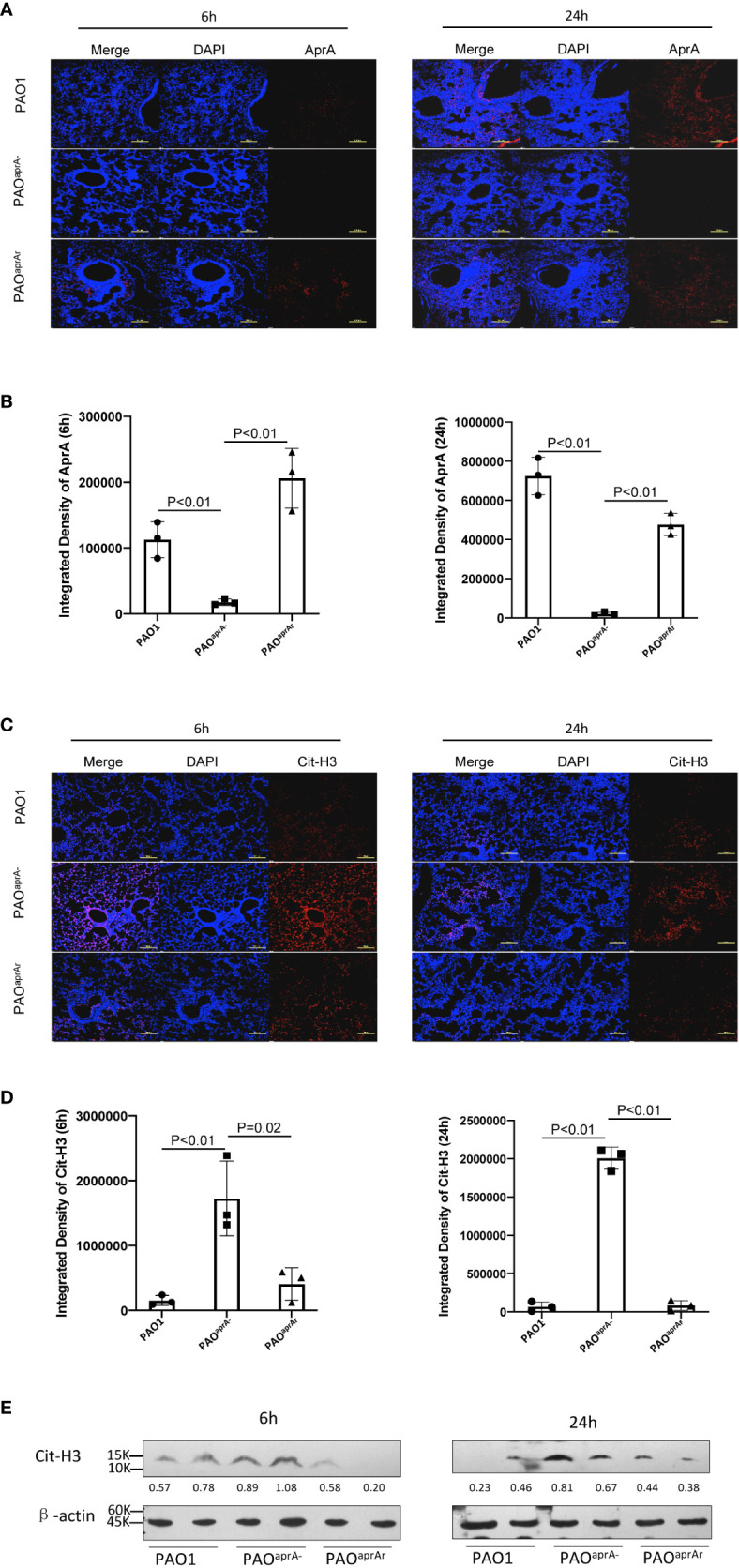
Deficiency of *aprA* increased NET levels *in vivo*. **(A–D)** Confocal scanning laser microscopic analysis of AprA and Cit-H3 in lung tissues of the indicated groups of mice (n = 10 per group). Lung tissue specimens were fixed at 6 h and 24 h post-challenge and stained using rabbit anti-AprA antibody **(A)** or rabbit anti-histone H3 (citrulline R2 + R8 + R17) antibody **(C)**, followed by a secondary tetramethylrhodamine (TRITC)-conjugated goat anti-rabbit antibody (red). Confocal laser microscopy, ×20. Scale bars, 100 μm. **(B, D)** Quantitation of immunofluorescence images shown in **(A, C)**. **(E)** The Cit-H3 levels in lung tissues of the indicated groups of mice were observed by western blotting. Lung tissues were homogenized at 6 h and 24 h post-challenge. The lysates were analyzed by western blotting using anti-β-actin and anti-histone H3 (citrulline R2 + R8 + R17) antibodies. All data are representative of three independent experiments. Data in **(B, D)** are represented as mean ± SD. Significant differences between groups were evaluated using two-tailed Student’s t tests.

As reported in previous studies, neutrophils are also able to release NETs to trap and kill bacteria (10, 11). We suspected that AprA secreted by *P. aeruginosa* may influence the level of NETs. First, we investigated whether AprA was detectable *in vivo* after mice were intratracheally challenged with *P. aeruginosa.* Lung tissue specimens were stained using specific antibody against AprA. As expected, we found that AprA was present in the lung tissues of mice challenged with PAO1 and PAO^aprA-^ (pAK1900-*aprA*), but not in mice challenged with PAO^aprA−^ (**Figures 2A, B**). We then determined the level of NETs in the lung tissues by staining for citrullinated histone H3 (Cit-H3), which is considered a hallmark of NETs (26, 27). Laser scanning confocal microscopy revealed that Cit-H3 levels were increased in the lung tissues of PAO^aprA−^-infected mice compared to those in PAO1-infected and PAO^aprA-^ (pAK1900-*aprA*)-infected counterparts (**Figures 2C, D**). NET levels were also measured by western blotting using an anti-Cit-H3 antibody. Consistent with the confocal microscopy results, it was demonstrated that NETs were more abundantly present in lung tissues of PAO^aprA–^infected mice than in lung tissues of the other two groups (**Figure 2E**).

### AprA Degraded NET Components but Did Not Inhibit NET Formation

**Figure 3:**
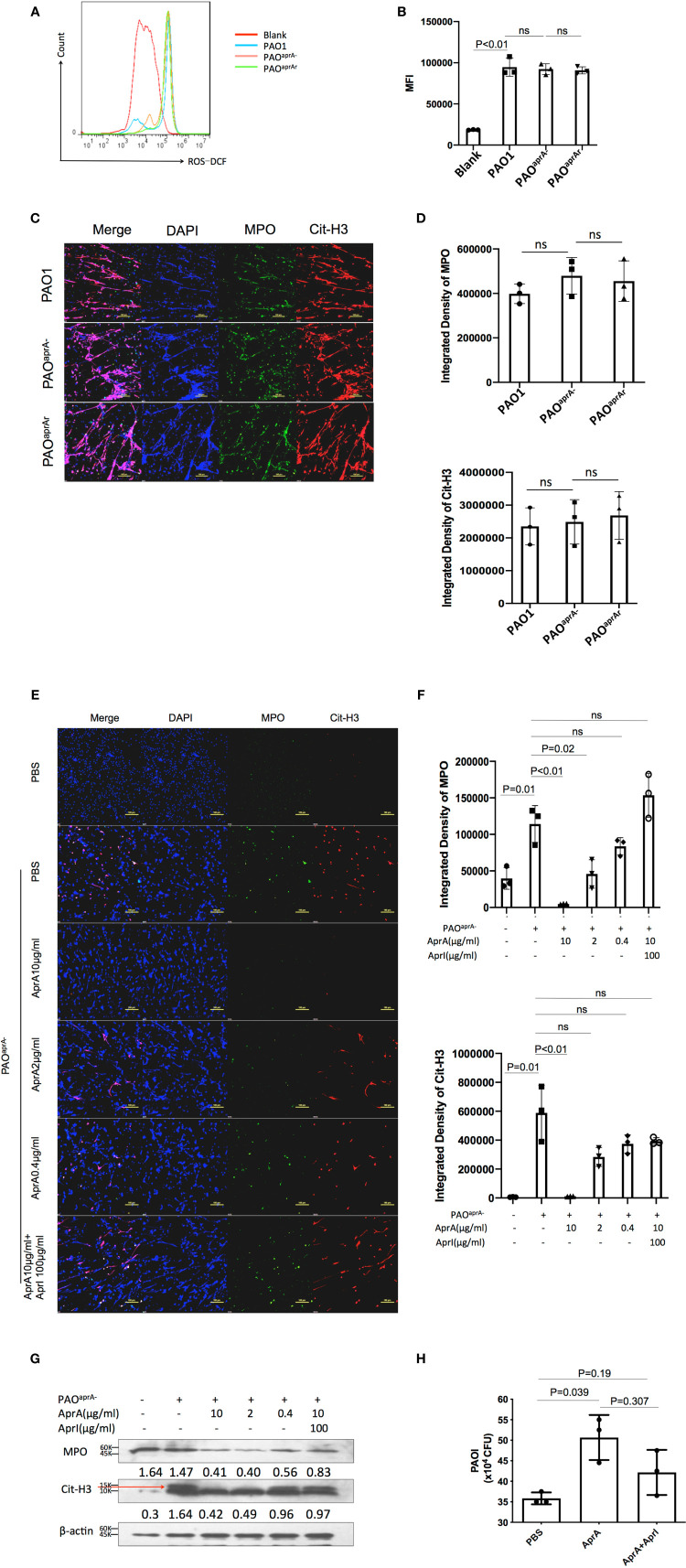
AprA degraded NET components but did not inhibit NET formation. Representative **(A)** or averages **(B)** of ROS production measured by flow cytometry (n=3). Human neutrophils were isolated and incubated with *P. aeruginosa* strains PAO1, PAO^aprA−^, or PAO^aprA-^ (pAK1900-*aprA*) (MOI=50). ROS production was detected using dichlorofluorescin dacetate (DCFH-DA). **(C, D)** Confocal scanning laser microscopic analysis of NET formation. **(C)** Neutrophils were isolated and stimulated with PAO1, PAO^aprA−^, or PAO^aprA-^ (pAK1900-*aprA*) (MOI = 50) for 3 h. Confocal laser microscopy, ×20. Scale bars, 100 μm. **(D)** Quantitation of immunofluorescence images shown in **(C)**. **(E–G)** Neutrophils were stimulated with PAO^aprA−^ (MOI = 50) for 3 h and then incubated with various concentrations of AprA and AprI for 2 h. NET components were then quantified by immunostaining **(E)** and western blotting **(G)**. Confocal laser microscopy, ×20. Scale bars, 100 μm. **(F)** Quantitation of immunofluorescence images shown in **(E)**. **(H)** The effect of AprA on NET-mediated bacterial killing. The surviving bacteria were counted, and the results normalized to the bacterial growth under the same conditions without neutrophils. All data are representative of three independent experiments. Data in **(B, D, F, H)** are represented as mean ± SD. Significant differences between groups were evaluated using two-tailed Student’s t tests ns, not significant.

To further investigate the mechanism of decreased NET levels *in vivo*, we first determined whether *aprA* deficiency affected NET formation. Previous studies have shown that reactive oxygen species (ROS) were released during *P. aeruginosa*-induced or phorbol myristate acetate (PMA)-induced NET formation (12, 28). Human neutrophils were isolated and incubated with *P. aeruginosa* (PAO1, PAO^aprA−^, or PAO^aprA-^ (pAK1900-*aprA*)) at a multiplicity of infection (MOI) of 50. ROS production was measured using flow cytometry, which showed that *P. aeruginosa*-triggered neutrophils released large amounts of ROS, but no significant difference was observed among the groups (**Figures 3A, B**). In addition, NET formation induced by the different *P. aeruginosa* strains was also detected by immunofluorescence. We found that PAO1, PAO^aprA−^, and PAO^aprA-^ (pAK1900-*aprA*) each stimulated NET formation in dose-dependent and time-dependent manners with no significant difference being observed among the strains (**Figures 3C, D**, **Figure S3A**, **B**). These results suggested that *aprA* deficiency had no significant effect on NET formation.

Considering that *aprA* deficiency induced elevated levels of Cit-H3, and AprA is a type of metalloprotease, we investigated whether AprA directly degraded Cit-H3 *via* its proteolytic activity. We first expressed and purified AprA, AprI, and flagellin in *Escherichia coli*. The activity of AprA was determined by incubation with flagellin in the presence and absence of AprI. Consistent with a previous study (29), AprA was found to efficiently degrade flagellin, which was blocked by AprI (**Figure S3C**). We then determined whether the purified AprA affected the formation of NETs. Human neutrophils were stimulated with PMA in the presence and absence of AprA. As expected, ROS production was not affected by AprA during PMA-induced NET formation (**Figure S3D**).

In further investigations, human neutrophils were incubated with PAO^aprA−^ or PMA for 3 h. Different concentrations of AprA were then added in the presence and absence of AprI. Components of NETs were stained using specific antibodies and detected by immunofluorescent labeling. The results of laser scanning confocal microscopy revealed that Cit-H3 was dramatically degraded by AprA in a concentration-dependent manner and this proteolytic activity of AprA was neutralized by AprI. Moreover, we found that MPO, another major component of NETs, was also degraded by AprA (**Figures 3E, F**, **Figure S3E**). These findings were confirmed by western blotting (**Figure 3G**). It has been demonstrated previously that histone H3 is citrullinated by peptidylarginine deiminase 4 (PAD4) during the formation of NETs (26, 27). Given our observed degradation of Cit-H3, we investigated whether AprA degraded only the modified form of histone H3. Histones from HL-60 cells were extracted and incubated with AprA. It was determined that AprA also efficiently degraded non-citrullinated histone H3. Moreover, we observed that other histones (H2A, H2B, and H4) were also degraded by AprA in a time-dependent manner (**Figure S3F–H**).

As histones and MPO have been previously shown to play an important role in NET antimicrobial activity (11), we examined whether the addition of AprA affected NET-mediated bacterial killing. We determined that the presence of AprA attenuated the bactericidal ability of NETs, which tended to be neutralized by AprI, although it did not reach a statistically significant difference (**Figure 3H**).

### NETs Played an Important Role in Murine Lung Infection Caused by *P. aeruginosa*

**Figure 4:**
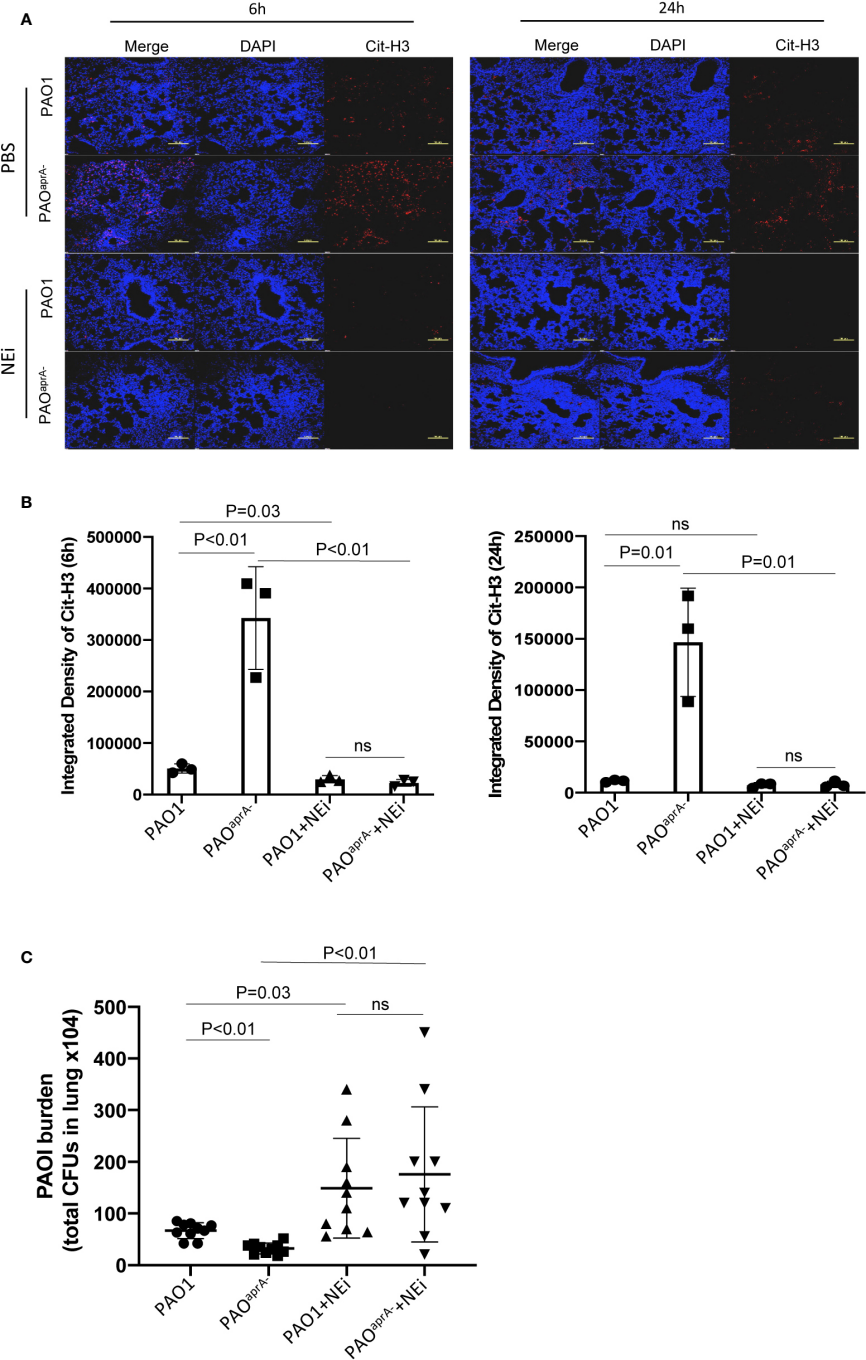
NETs play an important role in murine lung infection caused by *P. aeruginosa*. **(A)** Confocal scanning laser microscopic analysis of Cit-H3 in lung tissues of the indicated groups of mice. Mice (n = 16 per group) were injected with NEi [50 μg per mouse) or PBS 12 h before *P. aeruginosa* infection and every 12 h post-challenge. Lung tissue sections were obtained at 6 h and 24 h post-challenge. Confocal laser microscopy, ×20. Scale bars, 100 μm. **(B)** Quantitation of immunofluorescence images shown in **(A)**. **(C)** Bacterial burden in lung tissues of the indicated groups of mice. The number of bacteria in lung tissues was evaluated at 6 h post-challenge. All data are representative of three independent experiments. Data in **(B)** are represented as mean ± SD. Significant differences between groups were evaluated using two-tailed Student’s t tests. ns, not significant.

As demonstrated above, AprA effectively degraded NET components and attenuated NET-mediated bacterial killing. To assess the contribution of these findings in the pathogenesis of *P. aeruginosa*, we used neutrophil elastase inhibitor (NEi) to inhibit NET formation in the murine pneumonia model as described previously (20). We determined that NEi-treated mice exhibited a reduction in NET components compared to that of PBS-treated counterparts (**Figures 4A, B**).

The bacterial burden in lung tissues was also evaluated. Notably, the administration of NEi increased the number of bacteria in the lung tissues. Although the bacterial burden was higher in the PAO1 group than in the PAO^aprA−^ group of PBS-treated mice, no statistically significant difference was observed between the two *P. aeruginosa* strains in NEi-treated mice (**Figure 4C**).

To further validate the direct effect of AprA in our mouse pneumonia model, mice were treated with AprA or PBS before infected with PAO^aprA-^. Consistent with our previous results, AprA could efficiently degraded NETs components, and accompanied with higher levels of bacterial burden. However, no significant difference was observed between the two groups with administration of NEi (**Figure S4A** and **B** ).

### Antibody-Dependent Enhancement (ADE) of *P. aeruginosa* Infection

**Figure 5:**
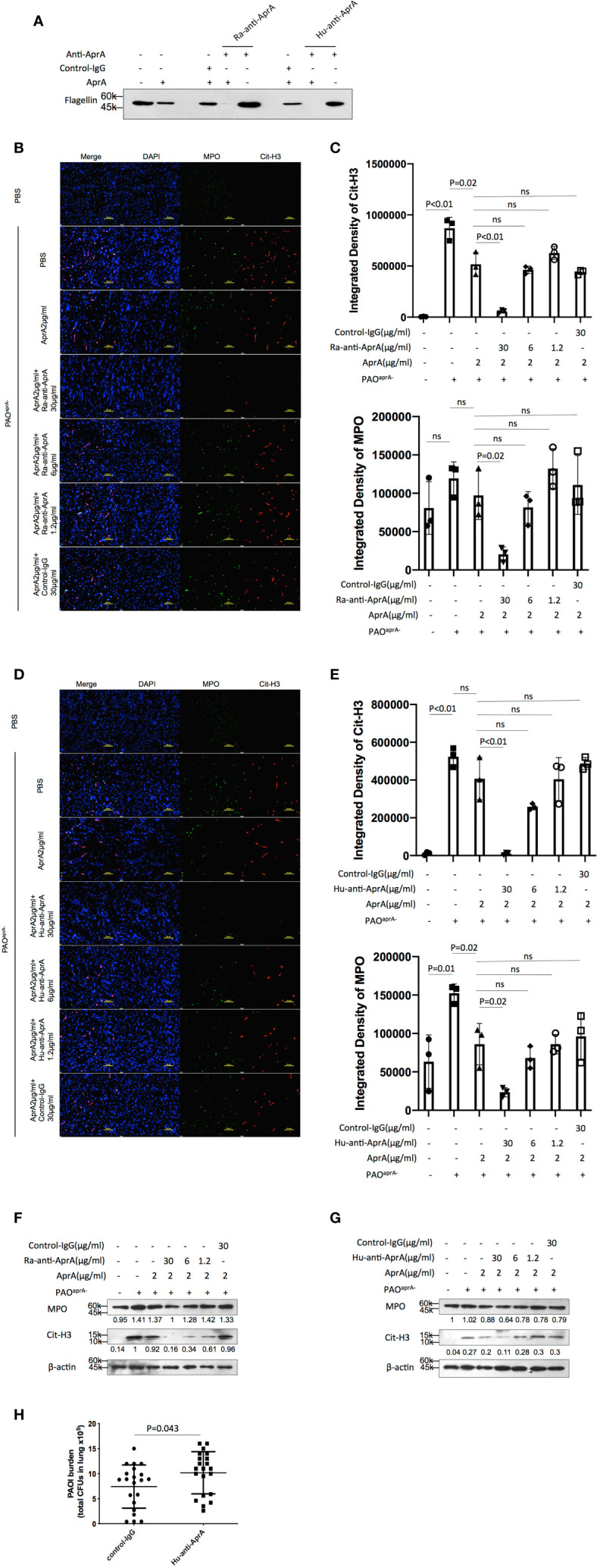
Antibody-dependent enhancement (ADE) of *P. aeruginosa* infection. **(A)** The effect of Ra-anti-AprA and Hu-anti-AprA on the degradation of flagellin mediated by AprA. The samples were analyzed by western blotting using anti-His antibody. **(B–G)** The effect of Ra-anti-AprA and Hu-anti-AprA on the degradation of NET components mediated by AprA. The samples were analyzed using a laser scanning confocal microscope **(B, D)** or western blotting **(F, G)**. Confocal laser microscopy, ×20. Scale bars, 100 μm. **(C, E)** Quantitation of immunofluorescence images shown in **(B, D)**. **(H)** Bacterial burden in lung tissues of the indicated groups of mice. Mice (n = 21 per group) were injected intraperitoneally with Hu-anti-AprA (100 μg per mouse) or control-IgG 24 h before PAO1 infection. The bacterial load in the lungs was calculated at 6 h post-challenge. All data are representative of three independent experiments. Data in **(C, E, H)** are represented as mean ± SD. Significant differences between groups were evaluated using two-tailed Student’s t tests. ns, not significant.

Since anti-AprA can be detected in patients with *P. aeruginosa* infection (30), we investigated whether anti-AprA could neutralize the degradation activity of AprA. First, we prepared rabbit polyclonal antibodies (Ra-anti-AprA) by immunization of the rabbit with AprA. The interaction between AprA and Ra-anti-AprA was evaluated by enzyme-linked immunosorbent assay (ELISA) and western blotting (**Figure S5A**, **C**). We then evaluated the effect of Ra-anti-AprA on the enzymatic activities of AprA. Surprisingly, we determined that Ra-anti-AprA did not inhibit, but rather enhanced, the degradation of flagellin by AprA (**Figure 5A**, **Figure S5E**). We then determined the effect of Ra-anti-AprA on the AprA-mediated degradation of NET components. After NET formation, Ra-anti-AprA increased the degradation of Cit-H3 and MPO in a dose-dependent manner in the presence of AprA (**Figures 5B, C, F**).

We then screened for the specific full human monoclonal antibody against AprA from human antigen binding fragment (Fab) libraries using phage display. After three rounds of screening, a specific antibody was generated and named Hu-anti-AprA. The interaction between AprA and Hu-anti-AprA was evaluated by ELISA and western blotting (**Figure S5B**, **D**). We then determined whether Hu-anti-AprA was able to neutralize the enzymatic activities of AprA. After incubation with Hu-anti-AprA, AprA-mediated degradation of flagellin and NET components was increased (**Figures 5A, D, E, G** and **Figure S5F**), suggesting that Hu-anti-AprA was also an agonist of AprA, similar to what was observed for Ra-anti-AprA.

To further assess the effect of Hu-anti-AprA in the murine pneumonia model using *P. aeruginosa*, mice were injected with Hu-anti-AprA or control-IgG 12 h prior to intranasal administration of PAO1. As expected, mice pretreated with Hu-anti-AprA exhibited increased numbers of bacteria in their lung tissues compared with those of the control mice (**Figure 5H**).

**Figure 6:**
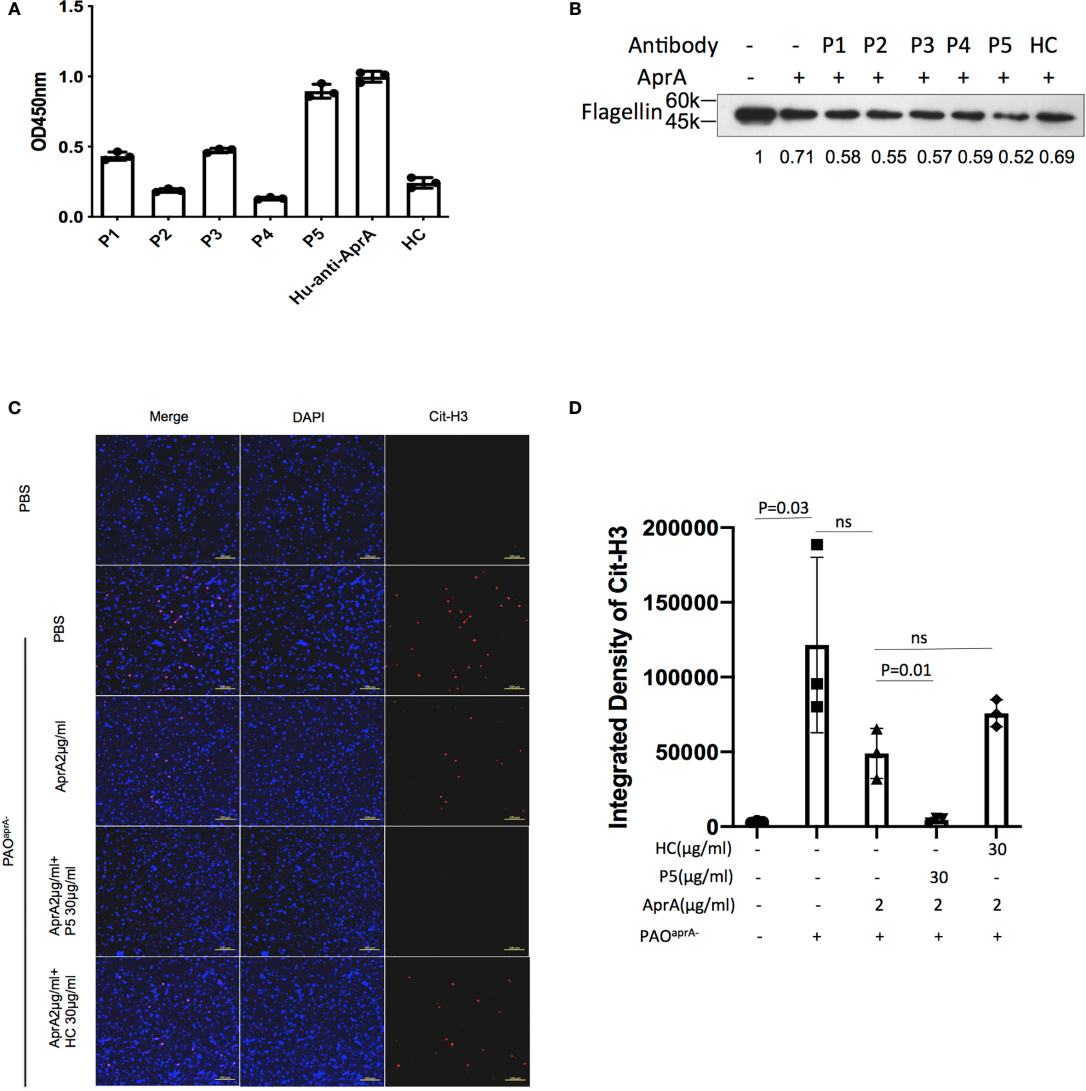
The effect of antibodies from patients with *P. aeruginosa* infection on AprA activities. **(A)** The levels of anti-AprA in P1 through P5 were detected by ELISA. AprA protein (10 μg/ml) was coated onto 96-well plates. P1 through P5 (10 μg/ml) were added to each well and then visualized using horseradish peroxidase (HRP)-conjugated goat anti-human IgG. **(B)** The effect of P1 through P5 on the degradation of flagellin mediated by AprA. The samples were analyzed by western blotting using anti-His antibody. **(C)** The effect of P5 on the degradation of NET components mediated by AprA. The samples were analyzed using a laser scanning confocal microscope. Confocal laser microscopy, ×20. Scale bars, 100 μm. **(D)** Quantitation of immunofluorescence images shown in **(C)**. All data are representative of three independent experiments. Data in **(A, D)** are represented as mean ± SD. Significant differences between groups were evaluated using two-tailed Student’s t tests. HC, healthy control. ns, not significant.

In subsequent studies, antibodies were purified from the individual sera of five patients with *P. aeruginosa* infection and named P1 through P5, respectively. The level of anti-AprA was determined by ELISA (**Figure 6A**). We then tested the effect of P1 through P5 on the enzymatic activities of AprA. We determined that P5 significantly enhanced the AprA-mediated degradation of flagellin, which correlated with its higher anti-AprA titer (**Figure 6B**, **Figure S5G**). Similar results were also observed for the AprA-mediated degradation of NET components (**Figures 6C, D**).

**Figure 7:**
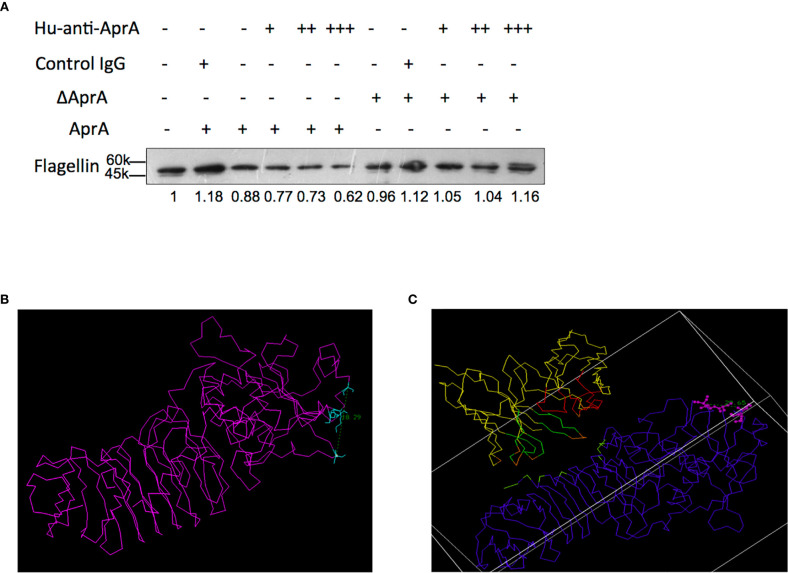
The potential mechanism of ADE effect of antibodies against AprA. **(A)** The effect of Hu-anti-AprA on the degradation of flagellin mediated by AprA or ΔAprA. The samples were analyzed by western blotting using anti-His antibody. **(B, C)** The distance of the flagellin binding pocket in AprA **(B)** or the complex of Hu-anti-AprA-AprA **(C)**. Data in **(A)** is representative of three independent experiments.

Based on the results given above, we speculated that ADE effect of anti-AprA was dependent on AprA enzyme activity. To test this hypothesis, we used the computer-guided homology modeling method and predicted the potential enzyme active sites (Ser90, Lys93, Ser138, Ser139 and Ser165) of AprA. Then we constructed and purified the mutant AprA protein (ΔAprA) with substitution of all the five amino acids with Ala. It was shown that the degradation of flagellin by ΔAprA was decreased compared with AprA (**Figure S5H**). In the further investigation, we detected whether Hu-anti-AprA enhanced the activities of ΔAprA. The results showed that Hu-anti-AprA recognized the ΔAprA as well as AprA (**Figure S5I**). However, compared with AprA, the enhancing activity of Hu-anti-AprA was disappeared when incubated with ΔAprA (**Figure 7A**).

To investigate how Hu-anti-AprA increased enzymatic activities of AprA, we constructed the complex structure of the Hu-anti-AprA-AprA. With the assistance of computer-guided homology modeling, the 3-D structures of the variable region of Hu-anti-AprA heavy and light chain (i.e. VH and VL domain) were modeled and optimized. After the 3-D structure model of the agonist antibody Fv fragment was constructed, the spatial complex structure of AprA and its agonist antibody Fv was obtained theoretically. The potential key domain and important residues identified by Hu-anti-AprA were predicted using the theoretical model (**Figure S6A**) and the key amino acids in antibody CDR (Arg11, Asp13, and Glu31) domain and AprA (Asn230, Thr231, Asn20, Ser12, Gln10, and Thr16) were demonstrated (**Figure S6B**).

Furthermore, we theoretically analyzed the conformational changes that occurred while the antibody binds to AprA. Based on the distance geometry method, the distance of the flagellin binding pocket in AprA (i.e., the distance between the main chain carbon atoms of Ser90 and Ser165) was calculated. When Hu-anti-AprA bound to AprA, the distance of the substrate-binding pocket changed from 18.29Å to 20.65Å (**Figures 7B, C**). These results suggested that the binding activity between AprA and flagellin could be strengthened when Hu-anti-AprA, the agonistic antibody, binding AprA. Furthermore, the solvent-accessible area of the binding pocket of AprA identified by its substrate was increased to about 82.5 Å2 when AprA bound to its agonist antibody.

## Discussion

Recently, NETs have been identified in several lung diseases associated with *P. aeruginosa*, suggesting that NETs can be induced *in vivo* after *P. aeruginosa* infection and may therefore contribute to host immune defenses (15–17, 31, 32). Previous studies reported that some bacteria have evolved strategies to escape being trapped and killed by NETs. For instance, by producing a nuclease, *Staphylococcus aureus* is able to degrade the DNA backbone and reduce its entrapment by NETs (33). In addition, Group A *Streptococcus* (GAS) is able to suppress NET formation by expressing streptococcal collagen-like protein 1 (Scl-1) (34). Other researchers have shown that *P. aeruginosa* isolated from patients with CF also has the ability to escape NET-mediated killing, but the precise mechanisms have not been elucidated (17). In the current study, we identified a potential mechanism for *P. aeruginosa* to escape NET-related immune clearance. Using a mouse pneumonia model, we determined that AprA levels were inversely correlated with NET components. Moreover, AprA was found to efficiently degrade Cit-H3 and MPO, which are known to coat DNA fibers to kill the entrapped bacteria, thereby suppressing the bactericidal ability of NETs *in vitro*. We also provided direct evidence that AprA-mediated degradation of NET components plays a critical role in lung infections caused by *P. aeruginosa*, which may partly explain the observation that AprA levels are negatively correlated with the prognosis of patients with *P. aeruginosa* infection.

Heterologous antigens can induce the activation of adaptive immunity to generate specific antibodies that typically neutralize the functions of the particular antigens. However, previous works reported that the detection of anti-AprA in patients with CF may help identify patients with aggressive forms of chronic infection (30, 35), which suggests that AprA-induced antibodies may be detrimental to the process of *P. aeruginosa* infection. Here, we demonstrated that anti-AprA in the serum of a rabbit immunized with AprA or in the serum of patients with *P. aeruginosa* infection increased the ability of AprA to degrade NET components. These results indicated that antibodies induced by AprA may enhance, not block, the activities of this enzyme. Moreover, we determined that AprA-specific antibody aggravated the infection caused by *P. aeruginosa* in the murine pneumonia model.

ADE of infection has been identified for numerous viruses, including Dengue virus (DENV), Ebola virus, human immunodeficiency virus, Zika virus, and measles virus (36–39). It has been determined in primary human macrophages that antibody-mediated cell entry of DENV enhances the fusion potential of the virus (40). However, the ADE of viral infection is obviously different from our current observations for *P. aeruginosa* infection in that the AprA-specific antibody in our study directly enhanced the activities of the antigen (AprA). Our results demonstrated that the ADE of anti-AprA was dependent on AprA enzyme activity. We speculated that the combination of AprA and anti-AprA could change the conformation of AprA, and make the enzyme active sites better exposed. More details of the enhancement of AprA activities mediated by Hu-anti-AprA will be investigated in the future.

In addition to flagellin, Cit-H3, MPO, and histones, previous studies have demonstrated that several proteins of the host immune system, such as complement C1q, C2, and C3, and cytokines like IFN-γ and TNF-α, are also substrates of AprA (29, 41, 42). Antibodies against AprA may also accelerate the degradation of these substrates and contribute further to the pathogenesis of *P. aeruginosa*. This will be investigated in future studies. Moreover, whether antibodies against other *P. aeruginosa* enzymes have similar ADE effects also needs to be addressed in the future.

**Figure 8:**
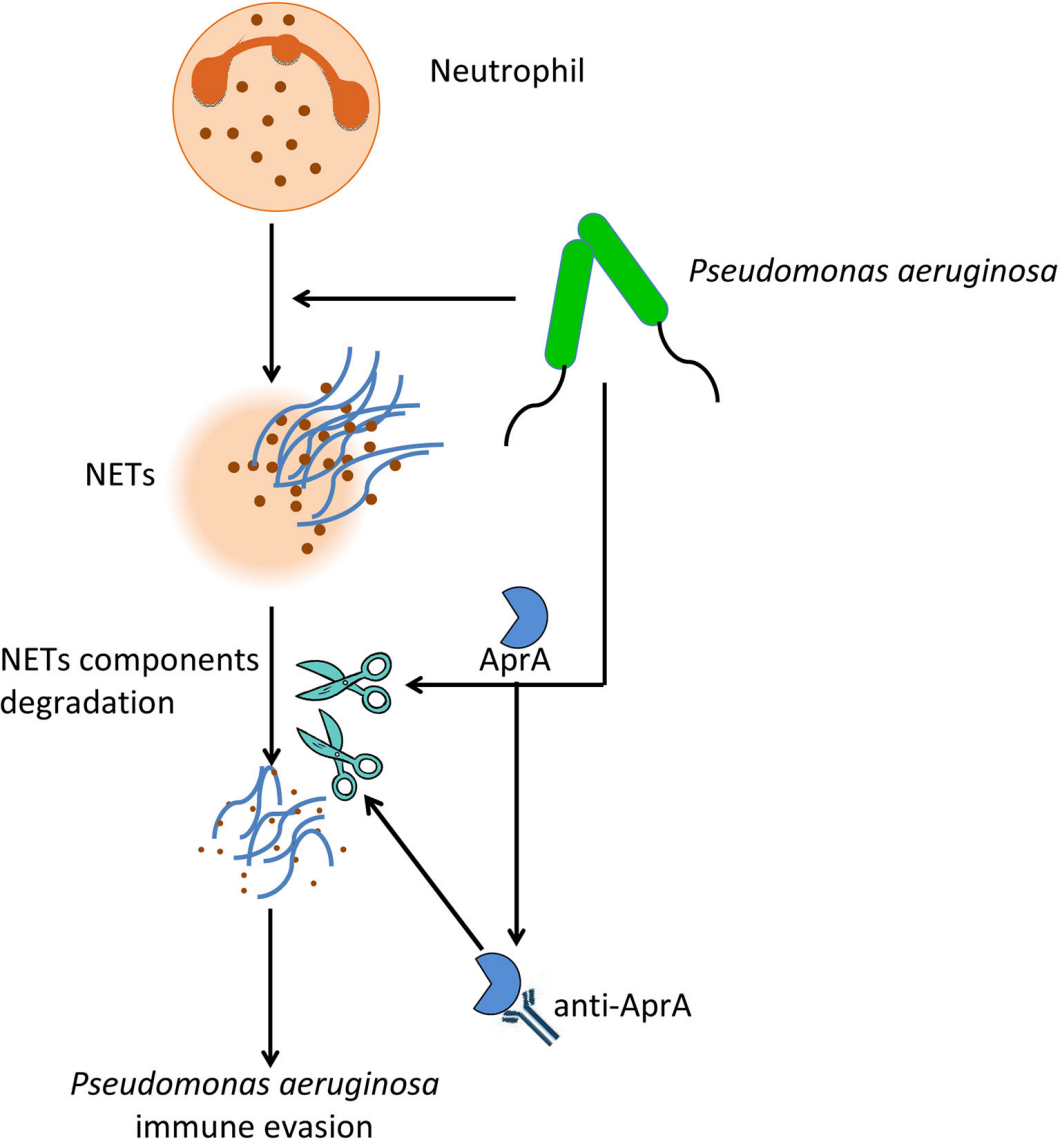
The effect of AprA on the pathogenesis of *P. aeruginosa*. *P. aeruginosa* can evade NETs killing by secreting AprA, which not only degrades NETs components, but also induce the production of specific antibodies that further enhance its effect.

In conclusion, our findings provided insight into two aspects of the effect of AprA on the pathogenesis of *P. aeruginosa* (**Figure 8**). First, AprA degraded NET components to help mediate immune evasion by *P. aeruginosa*. Second, AprA induced the production of antibodies that acted as agonists to enhance the activities of AprA and further exacerbate the *P. aeruginosa* infection.

## Data Availability Statement 

The raw data supporting the conclusions of this article will be made available by the authors, without undue reservation.

## Ethics Statement

The studies involving human participants were reviewed and approved by The ethics committee of the Peking University People’s Hospital. Written informed consent for participation was not required for this study in accordance with the national legislation and the institutional requirements.

## Author Contributions 

CJ acquired, analyzed, and interpreted data. YL and CL acquired and analyzed data. RF, RC, ZG, BX, YG, QW, and NY provided administrative, technical, and material support. YM, LL, JF, and BS supervised the study. YY and HW supervised the study and drafted the manuscript. GY conceived and designed the study, obtained funding and drafted the manuscript. All authors contributed to the article and approved the submitted version.

## Funding

This work was supported by grants from National Natural Science Foundation of China (http://www.nsfc.gov.cn) [81871618] and [31870127], and Shandong Provincial Major Scientific and Technological Innovation Project (MSTIP)[2019JZZY011012]. The funders had no role in study design, data collection and analysis, decision to publish, or preparation of the manuscript. The funders had no role in study design, data collection and analysis, decision to publish, or preparation of the manuscript.

## Conflict of Interest

The authors declare that the research was conducted in the absence of any commercial or financial relationships that could be construed as a potential conflict of interest.

## Abbreviations

AprA, alkaline protease; AprI, alkaline protease inhibitor; ADE, antibody-dependent enhancement; BALF, bronchoalveolar lavage fluid; CF, cystic fibrosis; COPD, chronic obstructive pulmonary disease; Cit-H3, citrullinated histone H3; MPO, myeloperoxidase; MOI, multiplicity of infection; NETs, neutrophil extracellular traps; NE, neutrophil elastase; NEi, neutrophil elastase inhibitor; PAD4, peptidylarginine deiminase 4; ROS, reactive oxygen species.
