# Supplementary material for: Antibodies Against Pseudomonas aeruginosa Alkaline Protease Directly Enhance Disruption of Neutrophil Extracellular Traps Mediated by This Enzyme
Source: Front Immunol. 2021 Mar 31;12:654649. doi: 10.3389/fimmu.2021.654649 (PMC8044376; doi:10.3389/fimmu.2021.654649)
Supplement: Supplementary file 1 [file DataSheet_1.pdf]

Fig. S1

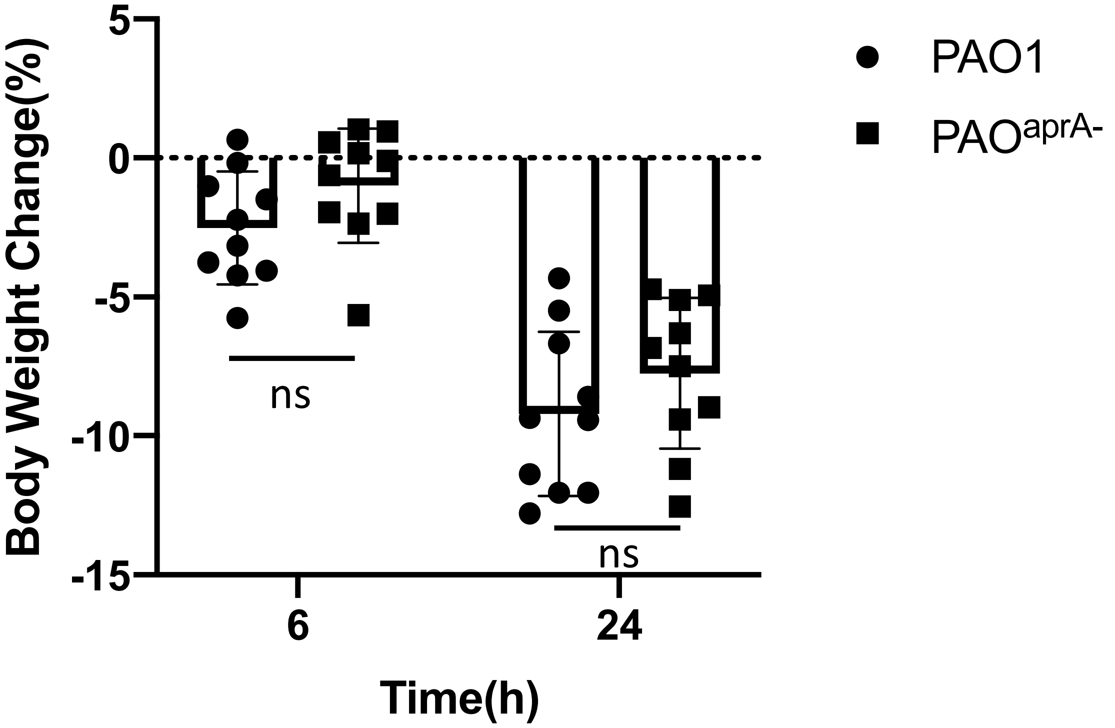

Fig. S1 Weight loss in mice during *P. aeruginosa* infection. The body weight was recorded at baseline, 6 h and 24 h post-challenge. Data are shown as the mean ± SD. Significant differences between groups were evaluated using two-tailed Student's t tests. ns, not significant.
