# Supplementary material for: Antibodies Against Pseudomonas aeruginosa Alkaline Protease Directly Enhance Disruption of Neutrophil Extracellular Traps Mediated by This Enzyme
Source: Front Immunol. 2021 Mar 31;12:654649. doi: 10.3389/fimmu.2021.654649 (PMC8044376; doi:10.3389/fimmu.2021.654649)
Supplement: Supplementary file 2 [file DataSheet_2.pdf]

Fig. S2

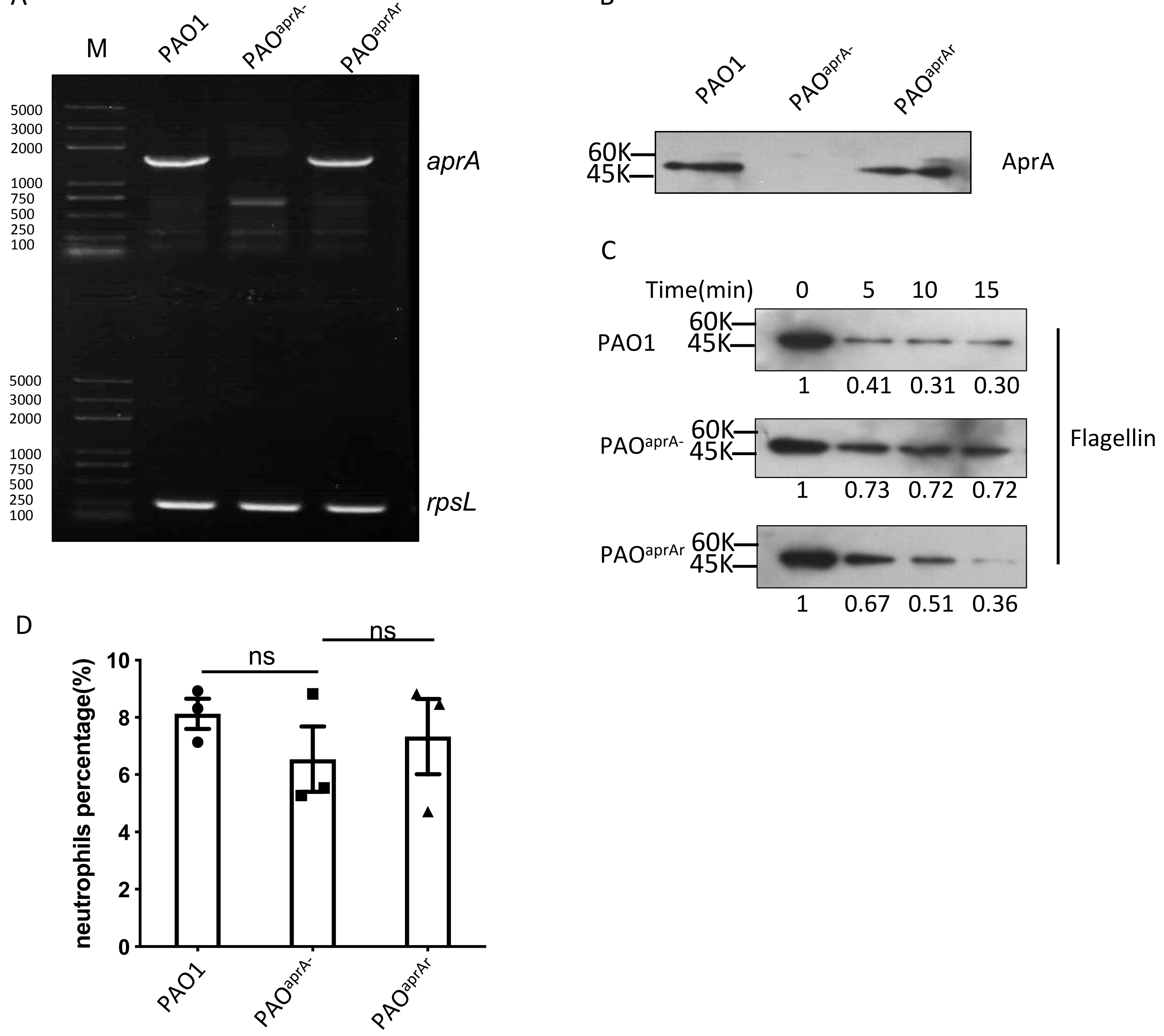

Fig. S2 Identification of the *aprA* rescued strain PAO^{aprAr} and the percentage of neutrophils in BALF.

(A) Expression of *aprA* was evaluated by detection of mRNA for the different *P. aeruginosa* strains. **(B)** AprA was detected in supernatant of different *P. aeruginosa* strains by western blotting using Ra-anti-AprA. **(C)** The supernatant of different *P. aeruginosa* strains were incubated with flagellin for a period of time. Then the samples were analyzed by western blotting using anti-His antibody **(D)** BALB/c mice (n = 3 per group) were challenged with a sub-lethal dose of PAO1, PAO^{aprA-}, and PAO^{aprAr}. The BALFs were collected at 6 h post-challenge and the percentage of neutrophils was evaluated using flow cytometry. All data are representative of three independent experiments. Data in (D) are represented as mean $\pm$ SD. Significant differences between groups were evaluated using Mann-Whitney test. ns, not significant.
