# Supplementary material for: Antibodies Against Pseudomonas aeruginosa Alkaline Protease Directly Enhance Disruption of Neutrophil Extracellular Traps Mediated by This Enzyme
Source: Front Immunol. 2021 Mar 31;12:654649. doi: 10.3389/fimmu.2021.654649 (PMC8044376; doi:10.3389/fimmu.2021.654649)
Supplement: Supplementary file 3 [file DataSheet_3.pdf]

Fig. S3

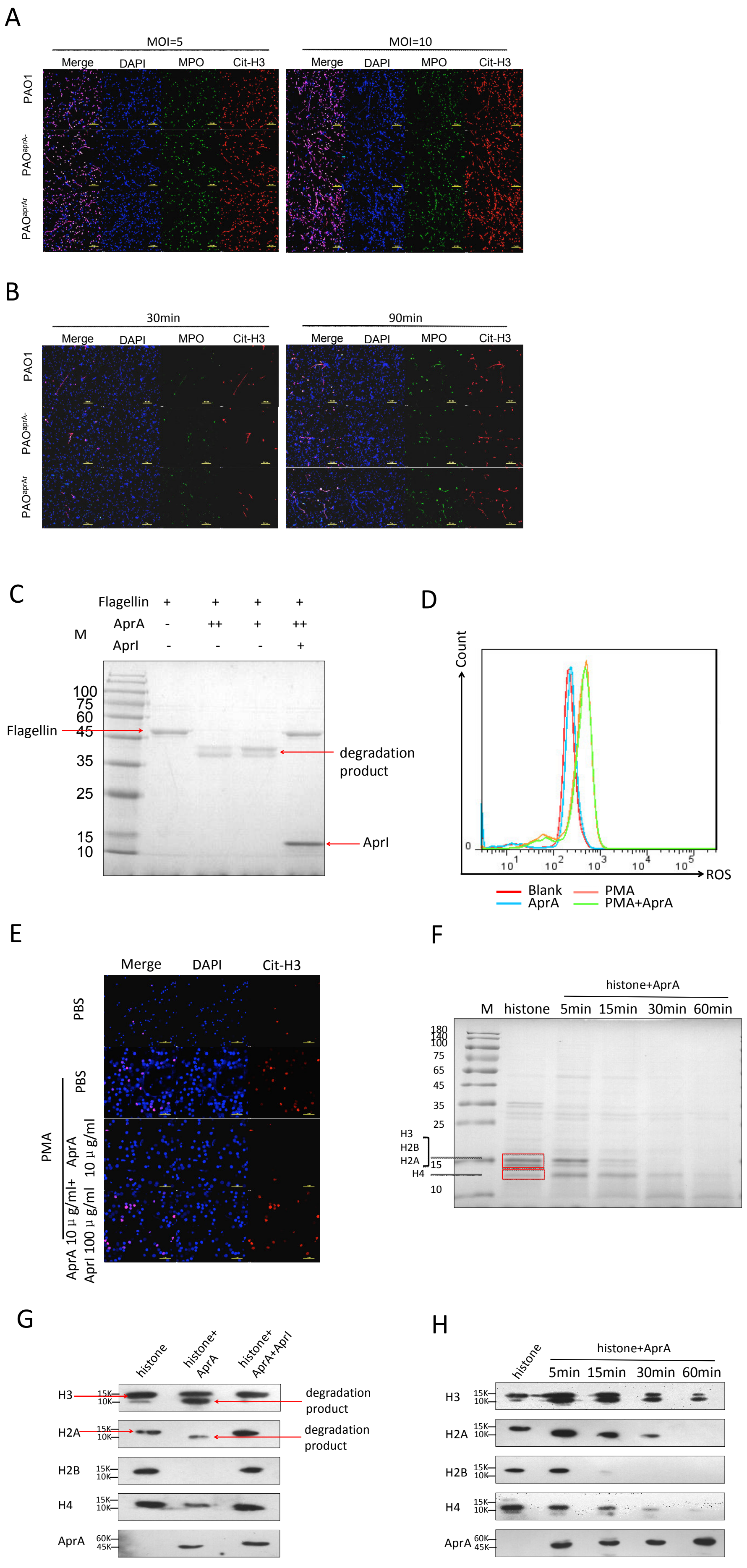
A
MOI=5
MOI=10
Merge
DAPI
MPO
Cit-H3
PAO1
B
30min
90min
C
Flagellin
AprA
AprI
M
100
75
60
45
35
25
15
10
degradation product
D
Count
ROS
Blank
AprA
PMA
PMA+AprA
E
PBS
PMA
F
histone
histone+AprA
5min
15min
30min
60min
H3
H2B
H2A
H4
G
histone+ AprA
histone+ AprA+AprI
AprA
H

Fig. S3 AprA degraded NET components but did not inhibit NET formation.

(A-B) Confocal scanning laser microscopic analysis of NET formation. Neutrophils were isolated and stimulated with PAO1, PAO^{aprA-}, or PAO^{aprAr} (MOI = 5, 10] for 3 h (A) or with PAO1, PAO^{aprA-}, or PAO^{aprAr} (MOI = 50] for 30 min and 90 min (B). Confocal laser microscopy, ×20. Scale bars, 100 μm. **(C)** AprA was incubated with flagellin in the presence or absence of AprI and analyzed by SDS-PAGE. **(D)** Neutrophils were stimulated with PMA in the presence or absence of AprA. The production of ROS was measured using flow cytometry. **(E)** Neutrophils were stimulated with PMA for 3 h and then incubated with various concentrations of AprA and AprI. NET components were then quantified by immunostaining. Confocal laser microscopy, ×40. Scale bars, 50 μm. **(F-H)** Histones were extracted from HL-60 cells and incubated with AprA in the presence or absence of AprI. The samples were then analyzed by SDS-PAGE (F) and western blotting (G, H). All data are representative of three independent experiments.
