# Supplementary material for: Antibodies Against Pseudomonas aeruginosa Alkaline Protease Directly Enhance Disruption of Neutrophil Extracellular Traps Mediated by This Enzyme
Source: Front Immunol. 2021 Mar 31;12:654649. doi: 10.3389/fimmu.2021.654649 (PMC8044376; doi:10.3389/fimmu.2021.654649)
Supplement: Supplementary file 4 [file DataSheet_4.pdf]

Fig. S4

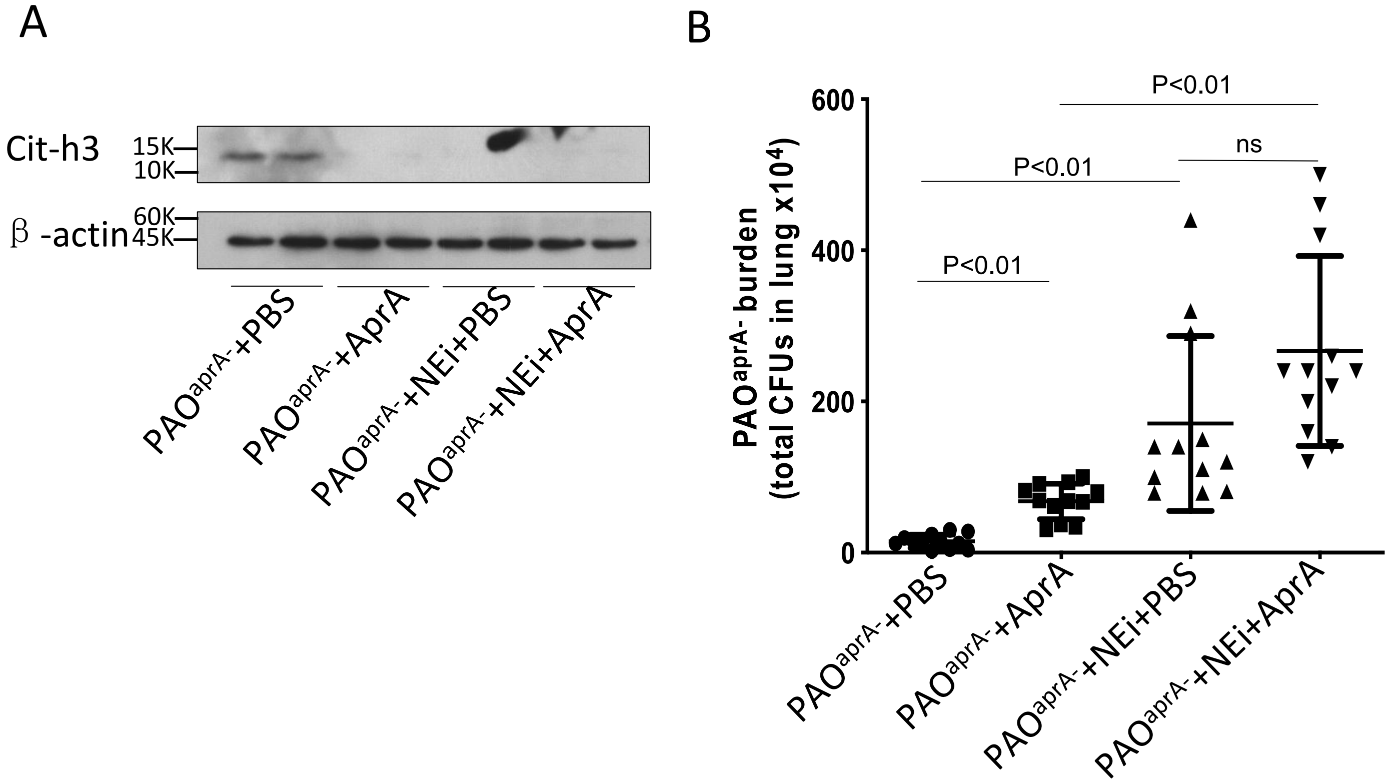


**Fig. S4 NETs play an important role in murine lung infection caused by *P. aeruginosa***

**(A)** The Cit-H3 levels in lung tissues of the indicated groups were observed by western blotting. Mice (n = 14 per group) were injected with NEi [50 μ g per mouse] or PBS as described above and inoculated intranasal with AprA [3 μ g per mouse] or PBS 30min before *P. aeruginosa* (PAO$^{aprA-}$) infection. Lung tissue sections were obtained and homogenized at 6 h post-challenge and analyzed by western blotting. **(B)** Bacterial burden in lung tissues of the indicated groups of mice. The number of bacteria in lung tissues was evaluated at 6 h post-challenge. All data are representative of three independent experiments. Data in (B) are represented as mean ± SD. Significant differences between groups were evaluated using two-tailed Student’s t tests. ns, not significant.
