# Supplementary material for: Antibodies Against Pseudomonas aeruginosa Alkaline Protease Directly Enhance Disruption of Neutrophil Extracellular Traps Mediated by This Enzyme
Source: Front Immunol. 2021 Mar 31;12:654649. doi: 10.3389/fimmu.2021.654649 (PMC8044376; doi:10.3389/fimmu.2021.654649)
Supplement: Supplementary file 5 [file DataSheet_5.pdf]

Fig. S5

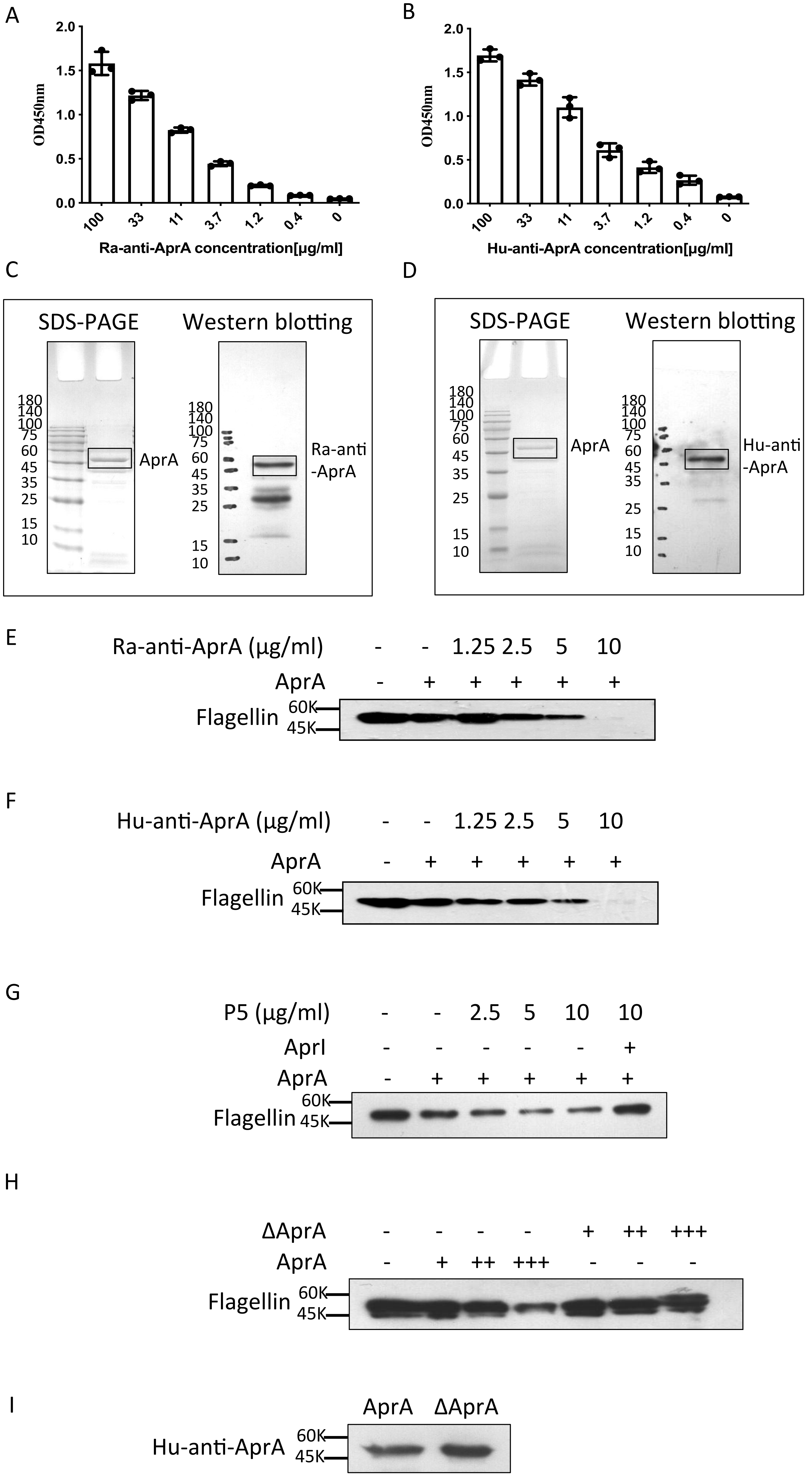
A
OD450nm
2.0
1.5
1.0
0.5
0.0
100
33
11
3.7
1.2
0.4
0
Ra-anti-AprA concentration[µg/ml]
B
OD450nm
Hu-anti-AprA concentration[µg/ml]
C
SDS-PAGE
Western blotting
180
140
100
75
60
45
35
25
15
10
AprA
Ra-anti
-AprA
D
SDS-PAGE
Western blotting
AprA
Hu-anti
-AprA
E
Ra-anti-AprA (µg/ml) - - 1.25 2.5 5 10
AprA - + + + + +
Flagellin
60K
45K
F
Hu-anti-AprA (µg/ml) - - 1.25 2.5 5 10
AprA - + + + + +
Flagellin
60K
45K
G
P5 (µg/ml) - - 2.5 5 10 10
AprI - - - - - +
AprA - + + + + +
Flagellin
60K
45K
H
ΔAprA - - - - + ++ +++
AprA - + ++ +++ - - -
Flagellin
60K
45K
I
AprA
ΔAprA
Hu-anti-AprA
60K
45K

Fig. S5 Anti-AprA accelerated the degradation ability of AprA. (A–D) The interaction of AprA and Ra-anti-AprA or Hu-anti-AprA was determined by ELISA (A, B) and western blotting (C, D). **(E–G)** The effect of Ra-anti-AprA (E), Hu-anti-AprA (F), and P5 (G) on the degradation of flagellin mediated by AprA. The samples were analyzed by western blotting using anti-His antibody. **(H)** The effect of AprA and ΔAprA on the degradation of flagellin. **(I)** The interaction of Hu-anti-AprA and AprA or ΔAprA was determined by western blotting. All data are representative of three independent experiments. Data in (A, B) are represented as mean ± SD.
