# Supplementary material for: Antibodies Against Pseudomonas aeruginosa Alkaline Protease Directly Enhance Disruption of Neutrophil Extracellular Traps Mediated by This Enzyme
Source: Front Immunol. 2021 Mar 31;12:654649. doi: 10.3389/fimmu.2021.654649 (PMC8044376; doi:10.3389/fimmu.2021.654649)
Supplement: Supplementary file 6 [file DataSheet_6.pdf]

Fig. S6

A B

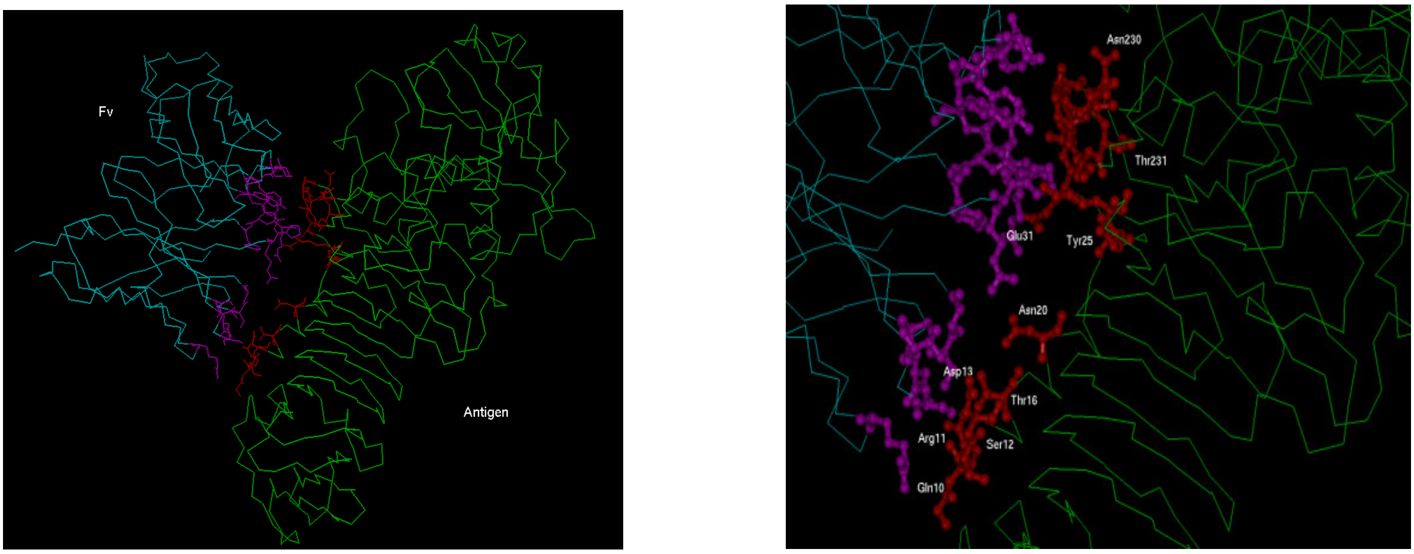

Fig. S6 The 3-D complex structure of AprA and Hu-anti-AprA based on a computer-guided molecular docking method.

(A) The 3-D complex structure of AprA and its antibody. The pale blue denoted the main chain carbon atom orientation of the Fv fragment of Hu-anti-AprA and the green indicated the main chain carbon atom orientation of AprA. The purple line represented the heavy atom orientation of the key residues in the antibody and the red marked the heavy atom orientation of the key residues in AprA. **(B)** The local 3-D binding domain structure of AprA and Hu-anti-AprA. The pale blue denoted the main chain carbon atom orientation of the Fv fragment of Hu-anti-AprA and the green represented the main chain carbon atom orientation of AprA. The purple ball and stick indicated the heavy atom orientation of the key residues in Hu-anti-AprA and the red ball and stick symbolized the heavy atom orientation of the key residues in AprA. The red balls and sticks marked the corresponding residues.
