# Supplementary material for: Antibodies Against Pseudomonas aeruginosa Alkaline Protease Directly Enhance Disruption of Neutrophil Extracellular Traps Mediated by This Enzyme
Source: Front Immunol. 2021 Mar 31;12:654649. doi: 10.3389/fimmu.2021.654649 (PMC8044376; doi:10.3389/fimmu.2021.654649)
Supplement: Supplementary file 7 [file Table_1.docx]

**Table S1. The characteristics of patients with *P. aeruginosa* infection.**

| No. of Patients | Age (year) | Gender | Diagnosis | Sample |
| --- | --- | --- | --- | --- |
| 1 | 45 | Male | Pulmonary infection | sputum |
| 2 | 63 | Male | Pulmonary infection | sputum |
| 3 | 29 | Male | Pulmonary infection | sputum |
| 4 | 62 | Male | Bloodstream infection | blood |
| 5 | 76 | Male | Pulmonary infection | sputum |
